# Use of Patient-Reported Data to Match Depression Screening Intervals With Depression Risk Profiles in Primary Care Patients With Diabetes: Development and Validation of Prediction Models for Major Depression

**DOI:** 10.2196/13610

**Published:** 2019-10-01

**Authors:** Haomiao Jin, Shinyi Wu

**Affiliations:** 1 Suzanne Dworak-Peck School of Social Work University of Southern California Los Angeles, CA United States; 2 Edward R Roybal Institute on Aging University of Southern California Los Angeles, CA United States; 3 Daniel J Epstein Department of Industrial and Systems Engineering University of Southern California Los Angeles, CA United States

**Keywords:** patient-reported data, patient-centered decision making, depression screening, depression, diabetes, health information technology, data analytics, predictive modeling, machine learning, data mining

## Abstract

**Background:**

Clinical guidelines recommend screening for depression in the general adult population but recognizes that the optimum interval for screening is unknown. Ideal screening intervals should match the patient risk profiles.

**Objective:**

This study describes a predictive analytics approach for mining clinical and patient-reported data from a large clinical study for the identification of primary care patients at high risk for depression to match depression screening intervals with patient risk profiles.

**Methods:**

This paper analyzed data from a large safety-net primary care study for diabetes and depression. A regression-based data mining technique was used to examine 53 demographics, clinical variables, and patient-reported variables to develop three prediction models for major depression at 6, 12, and 18 months from baseline. Predictors with the strongest predictive power that require low information collection efforts were selected to develop the prediction models. Predictive accuracy was measured by the area under the receiver operating curve (AUROC) and was evaluated by 10-fold cross-validation. The effectiveness of the prediction algorithms in supporting clinical decision making for six “typical” types of patients was demonstrated.

**Results:**

The analysis included 923 patients who were nondepressed at the study baseline. Five patient-reported variables were selected in the prediction models to predict major depression at 6, 12, and 18 months: (1) Patient Health Questionnaire 2-item score; (2) the Sheehan Disability Scale; (3) previous problems with depression; (4) the diabetes symptoms scale; and (5) emotional burden of diabetes. All three depression prediction models had an AUROC>0.80, comparable with published depression prediction studies. Among the 6 “typical” types of patients, the algorithms suggest that patients who reported impaired daily functioning by health status are at an elevated risk for depression in all three periods.

**Conclusions:**

This study demonstrated that leveraging patient-reported data and prediction models can help improve identification of high-risk patients and clinical decisions about the depression screening interval for diabetes patients. Implementation of this approach can be coupled with application of modern technologies such as telehealth and mobile health assessment for collecting patient-reported data to improve privacy, reducing stigma and costs, and promoting a personalized depression screening that matches screening intervals with patient risk profiles.

## Introduction

Depression is a common comorbid mental illness for many chronic conditions including diabetes [1-3]. About 10%-20% of adults with diabetes have major depressive disorders [1,2], but as high as 50% of these individuals are undiagnosed [3]. Comorbid depression and diabetes may significantly worsen the course of both disorders, leading to higher medical costs, reduced functioning and quality of life, increased risks of cardiovascular diseases, and increased mortality [4-9].

Depression screening is effective in identifying people with depression [10]. In primary care, validated tools such as the Patient Health Questionnaire (PHQ) are usually used for depression screening [11]. Results of the screening provide important information for decision making by clinicians and patients, often triggering referral of the patient to mental health professionals or an adjustment of care plans to better coordinate the physical and mental care.

Depression screening policy specifies the scope and frequency of screening and therefore plays a central role in implementing depression screening for large populations. Recent updates from the US Preventive Services Task Force (USPSTF) recommend depression screening for every adult, but they do not provide guidelines for screening frequency [10]. In practice, primary care providers may administer one-time depression screenings for all patients and must thereafter rely on practical strategies to determine the frequency [10,12].

One practical strategy recommends depression screening at a fixed frequency (eg, once a year). Providers may implement the fixed-frequency strategy by checking the time interval between the current clinical encounter and the last screening. They would administer a new screening if the time between screenings has been too long, according to a predefined requirement. This strategy has the benefit of guaranteeing at least a minimum screening frequency, but the major drawback is not differentiating between patients who are at high risk and those who are those at low risk. As a result, high-risk patients may receive insufficient screening and be undiagnosed for their depression, while low-risk patients may undergo unnecessary screening, which wastes clinical resources and patients’ time.

Another practical strategy relies on the judgements of health professionals (eg, primary care physicians, nurse practitioners, case managers, or social workers) to determine the screening frequency [12]. For example, a depression screening may be administered if a patient talks about his/her persistent depressed mood or loss of interest in performing daily activities with health providers. The problems associated with this strategy are threefold. First, a primary care encounter may be brief, and there would need to be enough time to address multiple conditions and issues along with depression-related problems [13]. Second, there are stigmas surrounding depression, which can often discourage, if not inhibit, patients from talking about their affective problems [14]. Third, culture differences may influence the expression of depression-related problems. Racial minorities such as Latinos may be more likely to attribute signs of depression to physical symptoms and not have their depression diagnosed [15,16].

Increasing the availability of patient-generated and patient-reported data may provide novel opportunities to improve policy making for depression screening. Many patient-generated and patient-reported variables such as socioeconomic status, stress level, and functional disability are significantly associated with depression [17-21]. Leveraging these data to develop accurate predictive models may improve identification of patients at high risk for depression and enable providers to match screening frequency with patient risk profiles. The systematic collection of patient-generated and patient-reported data also establishes a mechanism to encourage patients to communicate their affective problems: The logistics of a questionnaire serve as a formal protocol to elicit information, guiding patients to report their depression-related symptoms and concerns more effectively.

A few studies have explored the development of depression risk predictive tools [22-25]. King et al [22] used a stepwise logistic regression method to develop a risk prediction model for major depression at 6 and 12 months by using a dataset from general practice attendees. Huang et al [23] developed a logistic regression model for major depression at 6 and 12 months using electronic health record data. In addition, using the logistic regression method, Wang et al [24] developed a risk prediction model of major depression in 2-3 years for the general adult population. Finally, Liu et al [25] developed a decision tree model for predicting poststroke depression in stroke survivors. The predictive accuracy, as measured by the area under the receiver operating curve (AUROC) of those studies, was approximately 0.8.

The objective of this paper is to develop tools for predicting depression risk by using clinical, patient-generated, and patient-reported data from primary care patients with diabetes and discuss the implications of applying the prediction tools for making policies and practices for depression screening. The study will examine an array of demographic, clinical, patient-generated, and patient-reported variables and select the most predictive ones to assemble accurate depression prediction models. Implementation of the prediction models for risk prediction and the implications for patient data collection and decision making for the depression screening interval will be discussed.

## Methods

### Data Source

We analyzed data from the Diabetes–Depression Care-Management Adoption Trial (DCAT) [13,16,19,26-31], which enrolled 1406 patients with type 2 diabetes from eight safety-net primary care clinics affiliated with the Los Angeles County Department of Health Services (LAC-DHS), the second largest safety-net health care system in the United States. As described by Wu et al [13,16], the DCAT adopted a quasiexperimental comparative effectiveness design with three study groups to test an automated telephone depression screening and monitoring system, which was integrated with a diabetes disease management program in one group to facilitate the adoption of a collaborative depression care model. The other two groups were the LAC-DHS usual primary care clinics and the diabetes disease management program for adopting collaborative depression care, both of which did not include the automated telephone assessment system. The eight clinics were staffed by six teams of providers, two in each study group that were matched by geographic location and patient sociodemographics to form the three study groups. The patients were not randomly assigned; each patient was assigned to a study group based on the clinic from which he/she was recruited.

Patients were eligible for the DCAT if they were ≥18 years, had been diagnosed with type 2 diabetes, had a working phone number, spoke English or Spanish, and could read and understand the consent form. Patients with baseline possible suicidal ideation, cognitive impairment, alcohol abuse, or recent use of lithium or antipsychotic medication were ineligible for the trial. In all three trial groups, patients were assessed with the 9-item Patient Health Questionnaire (PHQ-9) [11], which has nine questions that are consistent with the nine criteria on which the 4th edition of the Diagnostic and Statistical Manual of Mental Disorders (DSM-IV) bases its diagnosis of depression. However, the PHQ-9 score was neither an inclusion criterion nor an exclusion criterion for trial enrollment. Hence, the DCAT sample comprised both patients with and those without depression. The trial was conducted from 2010 through 2013, with four waves of comprehensive assessments of patient-reported data through individual interviews at study baseline and 6-, 12-, and 18-month follow-ups.

### Study Sample

Our study sample featured patients with a 2-item Patient Health Questionnaire (PHQ-2) score<3 measured at study baseline [32,33]. PHQ-2 is a brief depression assessment tool with two questions that inquire about the frequency of depressed mood and anhedonia over the past 2 weeks [32,33]. The scores range from 0 to 6, and a cutoff point of 3 was shown to have a sensitivity of 83% and specificity of 92% for detection of current major depression [32]. The PHQ-2 comprises the first two items of the PHQ-9. The PHQ-9 score ranges from 0 to 27, and a cutoff point of 10 has been suggested for the diagnosis of major depression [11]. Kroenke et al [11,32] suggested a “two-step” procedure to administer PHQ screening in primary care: (1) All patients should receive the easy-to-administer PHQ-2 screening, and patients with PHQ-2 scores<3 are ruled out for current depression. (2) Patients with PHQ-2 scores≥3 will receive the full PHQ-9 assessment for diagnosis and determination of the severity of depressive symptoms. We used PHQ-9 scores≥10 at 6-, 12-, and 18-month follow-up assessments of DCAT as our primary predicted outcomes.

The reason that the subsample of the DCAT is pertinent for this predictive analysis study is because no intervention was provided for patients who were ruled out for depression at baseline. The only exception is that the patients in the technology group were reached by the automated call system every 3 months for up to 12 months to undergo assessment for the PHQ-2, pain, and self-management activities, including regular physical and fun activities and patient requests to be contacted by a provider. The call lasted about 2 minutes, and the response rate was about 50%. Because the DCAT provided no intervention and limited contact, these patients remained in their natural course of health conditions.

### Measurement of Patient-Generated and Patient-Reported Data

The predictive variables were selected from the extensive patient-reported data collected in the DCAT via in-person or telephone interviews, as described by Wu et al [16]. In addition to the PHQ-2 depressive symptomatology measurement described above, two standard questions from the structured clinical interview for DSM-IV were used to assess dysthymia [34]. The Sheehan Disability Scale was used to rate functional impairment on a 10-point Likert scale [35], which consisted of three questions on whether disease symptoms have disrupted the respondent’s work, social life, or family life. Chronic pain was defined as pain present most of the time for 6 months or longer during the past year and measured by the Short Form-12 [36] (one-item) pain impact questionnaire that asks respondents to rate the level of pain interference with normal work on a scale of 1-5 (1=*none* and 5=*extremely*). Anxiety was assessed by the Brief Symptom Inventory [37]. Health-related quality of life was assessed using the Medical Outcomes Study Short-Form Health Survey [36] and the Physical and Mental Component summaries. Patient satisfaction with diabetes care and emotional care was assessed with a single item on a 5-point Likert scale, ranging from very 1 (very dissatisfied) to 5 (very satisfied).

The assessment also included the Whitty 9-item questionnaire to assess diabetes symptoms [38], the brief two-item Diabetes Distress Scale to screen for distress [39], and the summary of Diabetes Self-Care Activities Questionnaire to assess self-reported adherence [40]. Moreover, we assessed social demographics, employment, self-reported weight and height (from which we calculated the body mass index), diabetes treatment and complications, comorbid medical illness, and socioeconomic stress.

### Statistical Analysis

To select the most predictive variables and develop the prediction models, we used the Least Absolute Shrinkage and Selection Operator (LASSO), a regression-based data mining technique that performs both variable selection and regularization to concurrently enhance the simplicity and prediction accuracy of the statistical model it produces [41,42]. LASSO can achieve both of these goals by introducing a penalization parameter, lambda, to a standard regression to penalize the size of coefficient estimation. As the value of lambda increases, every coefficient estimation shrinks toward 0 but at varying speed. The shrinkage speed provides a way to rank the predictive power of each variable because those that approach 0 slowly would have a better chance of being selected for the final prediction model when manipulating lambda from small values to large values. In this way, LASSO has the advantage of seeking a model that predicts well and is parsimonious. [42]

Candidate variables examined in this study included 53 demographic, clinical, and patient-generated and patient-reported variables measured at the baseline of DCAT (Table 1). We entered these variables into three LASSO logistic regressions to predict the occurrence of major depression, measured by PHQ-9≥10, at 6, 12, and 18 months. Thereafter, the variables were order-ranked based on their coefficient estimation shrinkage speed. We further considered efforts of patients required in reporting data for the variables. Hence, the criteria for selecting variables in the final predictive models were that the selected predictors should have slow shrinkage speed and be among the top ranked variables; in addition, those that require lesser efforts for patients to report data (measured by number of items in a scale) are preferred over those that required greater efforts.

We performed 10-fold cross-validation to evaluate the predictive accuracy of the candidate predictive models based on selected variables. The 10-fold cross-validation procedure randomly partitioned the dataset into 10 subsets. The complete procedure involved 10 rounds of computation; in each round, 9 of the 10 subsets of data were used to fit the prediction model and the remaining subset was used to assess predictive accuracy, which was measured by the AUROC. The 10 rounds of evaluation generated 10 AUROC scores, and the overall predictive accuracy was computed by taking the average. The resulting AUROC has a range of 0-1, with a larger value indicating better predictive accuracy. We also evaluated the model calibration using the Brier score. Model calibration refers to whether the predicted probabilities or scores can be used to predict the actual class membership probabilities. The Brier score has a range from 0 to 1, with smaller values indicating better model calibration. We examined the predictive models using the selected variables to predict major depression at 6, 12, and 18 months. The derived risk prediction algorithms can be interpreted in the same way as ordinary logistic regression, that is, the linear models compute the log odds risk of major depression. The final risk scores were calculated by taking the exponential of the linear scores. The final scores have a range from 0 to positive infinite, with higher scores indicating a higher risk of major depression. We evaluated sensitivity and specificity corresponding to different cutoff points of the predicted risk scores.

To demonstrate prediction algorithms’ effectiveness in supporting clinical decision making, we generated risk profiles for six “typical” types of patients: (1) the “median” patient, whose reported values in the selected predictors were set to the median values of our DCAT analysis sample; (2) the “average” patient, whose reported values in the selected predictors were set to the average values of our DCAT analysis sample; (3) patients with frequent diabetes symptoms, who reported experiencing a few days of diabetes-related symptoms (eg abnormal thirst, blurred vision, etc), determined by a Whitty-9 Diabetes Symptoms Scale score of 2.5, and had the rest of the predictors at the median values; (4) patients with some depressive symptoms, who had a PHQ-2 score of 2 and had the rest of the predictors at the median values; (5) patients who reported having previous depression problems but currently had no depressive symptoms; and (6) patients whose diseases affected their daily functioning, as measured by a Sheehan Disability Scale score of 4. We generated recommendations for clinical decision making based on the predicted depression risk scores for the 3 periods (ie, 6, 12, and 18 months) and chosen cutoff points. Depression screening was suggested for a period if the predicted depression risk score was equal to or higher than the chosen cutoff point. No screening was suggested for a period if the predicted depression risk scores up to that period were all lower than their cutoff points. If the predicted depression risk score for a period was lower than its cutoff point and at least one of the predicted risk scores for previous periods was higher than the other cutoff points, further clinical judgement was recommended to determine whether the patient needed depression screening. As shown below, we used a risk score of 8 as the cutoff point for the three periods. These cutoff points have sensitivities of 86%, 75%, and 90% and specificities of 64%, 71%, and 64% for 6, 12, and 18 months, respectively. Providers may choose different cutoff points based on their clinical needs. A lower cutoff point would increase sensitivity, reduce specificity, decrease the number of individuals with undiagnosed depression, and require more provider and patient time and resources to conduct more screenings.

## Results

We identified 999 patients with baseline PHQ-2 scores<3 from the DCAT dataset. We excluded 76 patients from analysis due to incomplete data on the candidate baseline predictors as listed in Table 1, and thus, 923 patients were included in the training and validation of risk prediction models. Table 1 summarizes the sample included in the analysis. Among the analysis sample, 83/776 (10.7%), 72/741 (9.7%), and 77/625 (12.3%) had PHQ-9 scores≥10 at 6, 12, and 18 months, respectively. The retention rates are 84.1% (776/923), 80.3% (741/923), and 67.8% (741/923) at 6, 12, and 18 months, respectively.

The comparison of baseline candidate predictors between patients who were later depressed at 6, 12, or 18 months and patients who were not later depressed is shown in the Multimedia Appendix 1. Table 2 shows that the LASSO regression method produced three predictive models to forecast major depression at 6, 12, and 18 months, respectively. Among the 53 variables examined, 6 patient-generated and patient-reported variables consistently appeared as the top predictors with the slowest shrinkage speed in all the three models: (1) the PHQ-2 score, measuring the two core symptoms of depression (ie, depressed mood and anhedonia); (2) the Sheehan Disability Scale score, measuring interference of health issues to work, social, and family life; (3) patient-reported previous problems with depression; (4) patient-reported diabetes symptoms; (5) patient-reported emotional burden from diabetes; and (6) patient-reported stressors, measuring the total number of stressors using a 12-item survey. The total number of stressors was excluded from the final models since the survey is longer than the other five scales and its exclusion has little impact on predictive accuracy. All the three final five-predictor models have an AUROC larger than 0.80. Sensitivity and specificity of identifying the depressed cases vary by cutoff points. Increasing sensitivity would reduce specificity, decrease the number of undiagnosed depression, and require more provider and patient time and resources to conduct more screenings. Balanced sensitivity and specificity are often recommended in the literature, but providers may opt to choose higher sensitivity if time and resources are sufficient. The primary goal is to reduce undiagnosed depression. The calibration performance of all the three models is good, as indicated by the small values of Brier scores.

Table 3 shows the predicted depression risk profiles and screening suggestions for five “typical” types of patients. The suggestions are based on predicted depression risk scores and the chosen cutoff points. Depression screening is suggested for a period if its predicted depression risk score is equal to or higher than the chosen cutoff point. No screening is suggested for a period if the predicted depression risk scores up to that period are all lower than their cutoff points. If the predicted depression risk score for a period is lower than its cutoff point and at least one of the predicted risk scores for the previous periods is higher than its cutoff points, further clinical judgement is suggested to determine whether the patient needs depression screening. Based on a cutoff point of 8 for the three prediction periods, patients with median reported values in the five predictors in the DCAT samples had depression risk scores lower than the cutoff points in all three periods and were therefore not recommended to receive screenings. Patients with average reported values in the five predictors, frequent diabetes symptoms, or some depressive symptoms were predicted to be at risk for depression at 6 months. They were advised to receive a follow-up screening at 6 months, and providers were recommended to consider whether further screenings at 12 and 18 months were warranted. Depression risk for patients who reported having previous depression problems but currently had no depressive symptoms was predicted to increase with time and across the cutoff points at 12 and 18 months. Thus, those patients were recommended screening at 12 and 18 months. Finally, patients who reported that their daily functioning was impaired by health status were predicted to be at an elevated risk for depression in all three periods. Depression screenings were recommended every 6 months for those high-risk patients. Providers may choose cutoff points other than 8, based on their clinical needs. A lower cutoff point would increase sensitivity, reduce specificity, decrease the number of individuals with undiagnosed depression, and require more provider and patient time and resources to conduct more screenings.

**Table 1:** Baseline data from the Diabetes–Depression Care-Management Adoption Trial to train and validate the depression prediction models.

Variables		Value (n=923)
**Demographic variables**		
	Age, mean (SD)	53.15 (9.52)
	Female, n (%)	557 (60)
	Latino, n (%)	818 (89)
	Birth place in the United States, n (%)	120 (13)
	Spanish the preferred language, n (%)	759 (82)
	Less than high school education, n (%)	629 (68)
	Married, n (%)	515 (56)
**Patient-generated and patient-reported variables**		
	Two-item Patient Health Questionnaire, mean (SD)	0.62 (0.80)
	Smoking, n (%)	62 (7)
	Onset age of diabetes, mean (SD)	43.27 (10.49)
	Family history of diabetes, n (%)	704 (76)
	Diabetes self-care score, mean (SD)	4.48 (1.25)
	Diabetes symptoms score, mean (SD)	1.50 (0.50)
	Chronic pain, n (%)	163 (18)
	Pain impact on normal work, n (%)	128 (14)
	Pain impact on social life, n (%)	72 (8)
	Bothered by thinking or dreaming of terrible things, n (%)	75 (8)
	Six-item Brief Symptom Inventory, mean (SD)	0.50 (1.82)
	Previous diagnosis of major depression, n (%)	41 (4)
	Previous diagnosis of anxiety disorders, n (%)	7 (1)
	Ever had a problem with depression, n (%)	128 (14)
	Ever had a problem with anxiety, n (%)	31 (3)
	Talking to someone about your depression, n (%)	44 (5)
	Number of stressors, mean (SD)	2.04 (2.07)
	Diabetes emotional burden, mean (SD)	2.56 (1.91)
	Diabetes regimen distress, mean (SD)	2.45 (1.89)
	Unemployed, n (%)	579 (63)
	Doing work for extra income, n (%)	85 (9)
	No health insurance, n (%)	68 (7)
	Feeling that my financial situation is getting worse, n (%)	288 (31)
	Having difficulty in paying bills, n (%)	615 (67)
	Having money left over at the end of the month, n (%)	839 (91)
	Financial worry score, mean (SD)	3.69 (2.07)
	Sheehan disability scale, mean (SD)	1.21 (2.12)
**Clinical variables**		
	Hemoglobin A_1c_, mean (SD) (%)	9.21 (2.13)
	Body mass index, mean (SD) (kg/m^2^)	32.62 (7.19)
	Number of diabetes complications, mean (SD)	1.11 (1.08)
	Taking insulin, n (%)	493 (53)
	On diabetes treatment - oral medication, n (%)	803 (87)
	On diabetes treatment - nutritionist observation, n (%)	32 (4)
	Had microalbumin test done in the past 6 months, n (%)	688 (75)
	Taking pain medication, n (%)	131 (14)
	Taking antidepressant, n (%)	53 (6)
	Taking anxiety medication, n (%)	7 (1)
	Number of ICD-9^a^ diagnoses in the past 6 months, mean (SD)	6.93 (4.27)
	Hospitalized overnight in past 6 months, n (%)	122 (13)
	ICU^b^ admission in the past 6 months, n (%)	23 (3)
	ER^c^ use in the past 6 months, n (%)	228 (25)
	Number of primary care visits in the past 6 months, mean (SD)	9.09 (5.94)
	Had missed medical appointment in the past 6 months, n (%)	92 (10)
	Future health care cost, mean (SD)	6614.79 (3714.58)
	Enrolled into disease management program, n (%)	643 (70)
	Receiving automatic telephone screening and monitoring, n (%)	307 (33)

^a^ICD-9: International Classification of Diseases, Ninth Revision, Clinical Modification.

^b^ICU: intensive care unit.

^c^ER: emergency room.

**Table 2:** Predictive accuracy to forecast major depression at 6, 12, and 18 months among primary care patients with diabetes recruited in the Diabetes–Depression Care-Management Adoption Trial study.

Predicted outcome	Algorithm to predict log odds risk score of major depression	AUROC^a^	Brier score	Cutoff^b^	Sensitivity (%)	Specificity (%)
6-month depression	100×exp(–4.58 + 0.55×PHQ2^c^ + 0.13×SDS^d^ + 0.80×PBD^e^ + 0.80×DSS^f^ + 0.09×DEB^g^)	0.80	0.07	22; 14; 8; 5	49; 70; 86; 90	88; 79; 64; 45
12-month depression	100×exp(–4.83 + 0.54×PHQ2 + 0.21×SDS + 1.26×PBD + 0.80×DSS + 0.05×DEB)	0.81	0.06	22; 14; 8; 5	56; 67; 75; 86	90; 84; 71; 56
18-month depression	100×exp(–4.53 + 0.46×PHQ2 + 0.16×SDS + 1.23×PBD + 0.62×DSS + 0.20×DEB)	0.83	0.07	22; 14; 8; 5	67; 76; 90; 91	87; 78; 64; 47

^a^AUROC: area under receiver operating curve.

^b^The cutoff points 22, 14, 8, and 5 correspond to 100×exp(–1.5), 100×exp(–2.0), 100×exp(–2.5), and 100×exp(–3.0), respectively.

^c^PHQ-2: Patient Health Questionnaire - 2-item.

^d^SDS: Sheehan Disability Scale.

^e^PBD: ever had a problem with depression.

^f^DSS: Diabetes Symptoms Scale.

^g^DEB: diabetes emotional burden.

**Table 3:** Examples of patient depression risk profiles and suggestions regarding follow-up depression screening.

Profile	PHQ-2^a^	SDS^b^	PBD^c^	DSS^d^	DEB^e^	Predicted depression risk score					
						6 mo		12 mo		18 mo	
						Score	Suggestion	Score	Suggestion	Score	Suggestion
											
The “median” patient	0	0	0	1.33	1	3	No Screening	2	No Screening	3	No Screening
The “average” patient	0.62	1.21	0.14	1.50	2.56	22	Depression Screening	6	Clinical judgement	8	Clinical judgement
Frequent diabetes symptoms	0	0	0	2.5	1	8	Depression Screening	6	Clinical judgement	6	Clinical judgement
Some depressive symptoms	2	0	0	1.33	1	10	Depression Screening	7	Clinical judgement	8	Clinical judgement
Had previous depression problems but currently no symptoms	0	0	1	1.33	1	7	No Screening	9	Depression Screening	10	Depression Screening
Diseases affect functionality	1	4	0	2.5	1	25	Depression Screening	25	Depression Screening	19	Depression Screening

^a^PHQ-2: Patient Health Questionnaire - 2-item.

^b^SDS: Sheehan Disability Scale.

^c^PBD: ever had a problem with depression.

^d^DSS: Diabetes Symptoms Scale.

^e^DEB: diabetes emotional burden.

## Discussion

### Principal Findings

This paper examined 53 demographic, clinical, and patient-generated and patient-reported variables from a large clinical trial dataset generated from an urban safety-net primary care setting. Using a regression-based data mining technique, five predictive variables, which are all patient-generated and patient-reported, were selected to develop three accurate prediction models to forecast major depression at 6, 12, and 18 months.

Compared with the fixed-frequency depression-screening policy, the prediction models enable providers to distinguish patients at high risk from those at low risk for depression and match screening frequency to patient risk profiles. The results recommend providers, who would follow USPSTF’s guidelines to conduct depression screening for every adult patient, to collect additional patient-generated and patient-reported data from patients judged to be nondepressed; these data included information on functional disability, whether the patient ever had problems with depression, diabetes symptoms, and diabetes emotional burden. The risk scores computed from the initial depression-screening result and the additionally collected patient-generated and patient-reported data could provide valuable decision-support information for providers.

Compared with the clinical judgement–based depression-screening policy, the selected predictors specify a formal protocol to elicit targeted and useful information about depression risks. Rather than the occasion and less structured way of talking about depression during clinical encounters, a formal data collection protocol can guide patients to systematically review and disclose indicators of depressive vulnerabilities, such as current depressive symptoms and ever having had problems with depression, and stressors that may activate depression in the future like functional disability, various diabetes symptoms, and emotional burden from diabetes. Additionally, providers may carry out the predictor collection protocol in private and confidential ways, such as through a technology-facilitated assessment [31], to reduce patient stigma and encourage the disclosure of depression-related information.

It is worth mentioning that having clinical resources in place to ensure appropriate information collection and patient outreaching/follow-ups is indispensable for successful implementation of the prediction model–based screening policy. In the United States, such clinical resources are often implemented in the form of team-based collaborative depression care in a primary care setting [43,44], which can be effective but costly, especially for safety-net care systems [27]. Recent developments in telehealth and mobile health technologies for depression, such as automated telephone [31,45] and text-messaging [46] depression assessment, may facilitate more cost-effective implementation. Hay et al [27] reported that using an automatic telephonic assessment technology can reduce the costs of collecting information on depressive symptoms, medication, and functioning from US $35 per assessment by a health professional to US $2 per assessment via technology. Such automatic telephonic technology was also reportedly perceived as useful, private, and secure by safety-net primary care patients [29]. Patient-generated and patient-reported predictors for depression can also be collected via a text messaging assessment, which lowers costs even more than an automatic telephonic assessment [46]. In addition, providers may use technologies to proactively reach out to patients identified as high risk by the prediction models. Wu et al [13] reported that a proactive collaborative primary and depression care intervention based on automatic telephonic assessment is effective in reducing depressive symptoms, facilitating the diabetes care processes, and improving patient satisfaction.

Findings from the predictive modeling analysis in this paper may benefit providers from countries other than the United States that recommend a different approach for managing depression screening. The UK National Institute for Health and Care Excellence recommends targeted case identification rather than universal depression screening [47]. The Canadian Task Force on Preventive Health Care currently does not provide strong recommendations for depression screening but advises that clinicians be alert to the possibility of depression in patients with clinical clues, especially those at increased risk of depression, and implement treatment as appropriate when depression is diagnosed [48]. Findings of this paper identify top predictors for depression among patients with diabetes and suggest profiles of diabetic patients at an increased risk of depression. High-risk patients may include patients with frequent diabetes symptoms; patients with previous problems with depression; patients with some but not clinically significant depressive symptoms; and patients whose daily functioning in work, social, and family life is impaired by diseases.

### Limitations

This study has several limitations. The analysis is based on a clinical trial dataset consisting of safety-net primary care patients with type 2 diabetes. Whether the results could be generalized to other patient populations with diabetes needs more validation. An advantage of the data mining technique used in this study is that it could be automated. This eases repetition of the current analysis on other datasets. This study is also limited by measuring the major depression by PHQ-9, which is a depression screening tool rather than a diagnostic tool. The use of scores of ≥10 to classify major depression in this paper may lead to false-positive depression cases. As described in Kroenke et al [49], PHQ-9<10 seldom occur in individuals with major depression, whereas scores of ≥15 usually signify the presence of major depression. In the gray zone of scores between 10 and 14 points, increasing the PHQ-9 scores is associated with increasing specificity and declining sensitivity. This paper tested only one predictive algorithm—LASSO. Although the algorithm satisfactorily achieved our goals of developing parsimonious prediction models with comparative predictive accuracy, other data mining algorithms may achieve similar or even better performance. Estimating standard errors or CIs and performing statistical tests are still challenging and unresolved issues for LASSO regression [50]. As a result, we are unable to show reliable estimates of the CIs for the LASSO estimates of regression coefficients. The dataset used to develop the prediction models covered an 18-month period, which may not be long enough to state recommendations about the screening periodicity. The DCAT intervention applied to the study sample may result in different relationships between the baseline predictors and future depression across different study groups, although our analysis has included the study group as one of the candidate predictors and did not determine this variable to be among the top predictive ones. Finally, there is a lack of external validation of the derived prediction models besides the cross-validation used in the paper. Future research should test the prediction models derived in this paper on an external dataset. It is also unknown whether primary care providers would accept use of the prediction models to guide policy making for depression screening. The success of actual implementation may be influenced by many factors such as predictive accuracy, leadership support, appropriate training, providers’ experience and expectation of predictive analytics, and the costs and benefits associated with the implementation. It may be burdensome and challenging for providers to collect the patient-generated and patient-reported data required to run the prediction models. Providers may also be overwhelmed by responding to high-risk depression cases identified by the prediction models. As discussed above, integrating the prediction models with telehealth and mobile-health technologies could automate the data collection, risk prediction and patient outreach, and consequently relieve providers from time-consuming work. Testing the feasibility and effectiveness of such a prediction- and technology-facilitated depression-screening intervention would be worth further investigation.

### Comparisons to Prior Works

This study adds to the growing body of research that utilizes predictive analytics to improve the forecasting of depression [19,22-25,28]. A strength of this study is that the derived prediction models use a relatively small number of predictors (ie, 5 compared with 10-20 in most prior studies) while still achieving comparable predictive accuracy as measured by the AUROC. This reduces the time and effort needed in clinical practices to collect necessary information for depression prediction and may therefore facilitate the implementation of the prediction model–based depression-screening policy. A second strength of this study is that it examined a broad array of candidate predictors that include both patient-generated and patient-reported variables and clinical variables. Finally, the prediction models are developed in a policy-making context and address an important policy issue (ie, unknown optimal depression screening intervals) as identified in the recent update of clinical guideline on depression screening from the USPSTF [10]. Surprisingly, few studies have addressed this important policy issue [10,12]. Our findings suggest that the collection of patient-generated and patient-reported data with the application of advanced data mining techniques may be a promising direction to match depression screening intervals with patients’ depression risk profiles.

### Conclusions

This study developed prediction models to improve identification of primary care patients with diabetes who are at high risk for depression and discusses the implications for policymaking on depression screening. The derived models rely on five patient-generated and patient-reported predictors to make accurate predictions. Implementation of the prediction models, especially when integrating with telehealth and mobile-health assessment technologies for data collection and patient outreach, may improve privacy, reduce stigma and costs, and promote a personalized depression-screening policy that matches screening frequency with patient risk profiles.

## Appendix

Multimedia Appendix 1 Comparison of baseline data from the Diabetes–Depression Care-Management Adoption Trial (DCAT) between patients who were later depressed versus those who were not depressed.

## Abbreviations

AUROC: area under the receiver operating curve
DCAT: Diabetes–Depression Care-Management Adoption Trial
DEB: diabetes emotional burden
DSM-IV: Diagnostic and Statistical Manual of Mental Disorders
DSS: Diabetes Symptoms Scale score
ER: emergency room
ICD-9: International Classification of Diseases, Ninth Revision, Clinical Modification.
ICU: intensive care unit
LAC-DHS: Los Angeles County Department of Health Services
LASSO: Least Absolute Shrinkage and Selection Operator
PBD: ever had a problem with depression
PHQ: Patient Health Questionnaire
SDS: Sheehan Disability Scale score
USPSTF: US Preventive Services Task Force
