# Supplementary material for: Use of Patient-Reported Data to Match Depression Screening Intervals With Depression Risk Profiles in Primary Care Patients With Diabetes: Development and Validation of Prediction Models for Major Depression
Source: JMIR Form Res. 2019 Oct 1;3(4):e13610. doi: 10.2196/13610 (PMC6774232; doi:10.2196/13610)
Supplement: Multimedia Appendix 1 [file formative_v3i4e13610_app1.pdf]

Supplemental Table. Comparison of Baseline Data from the Diabetes–Depression Care-Management Adoption Trial (DCAT) between Patients Who Were Later Depressed versus Who Were Not Depressed

**Baseline Variables**	**Patients Who Were Not Later Depressed (n=675)**	**Patients Who Were Later Depressed[a] (n=164)**	***P***
Demographic variables			
Age, mean (SD)	53.16 (9.76)	53.81 (7.88)	0.37
Female, No. (%)	414 (61.3)	97 (59.1)	0.67
Latino, No. (%)	597 (88.4)	151 (92.1)	0.23
Birth place in United States, No. (%)	597 (88.4)	137 (83.5)	0.12
Spanish the preferred language, No. (%)	558 (82.7)	137 (83.5)	0.88
Less than high school education, No. (%)	466 (69.0)	105 (64.0)	0.25
Married, No. (%)	376 (55.7)	92 (56.1)	0.99
Patient-Generated and Patient-reported variables			
Patient Health Questionnaire, 2-item (PHQ-2), mean (SD)	0.51 (0.75)	1.09 (0.83)	<.001
Smoking, No. (%)	47 (7.0)	7 (4.3)	0.28
Onset age of diabetes, mean (SD)	43.64 (10.53)	42.69 (9.72)	0.27
Family history of diabetes, No. (%)	512 (75.9)	133 (81.1)	0.18
Diabetes self-care score, mean (SD)	4.48 (1.24)	4.48 (1.31)	0.95
Diabetes symptoms score, mean (SD)	1.42 (0.42)	1.85 (0.60)	<.001
Chronic pain, No. (%)	100 (14.8)	49 (29.9)	<.001
Pain impact on normal work, No. (%)	79 (11.7)	41 (25.0)	<.001
Pain impact on social life, No (%)	36 (5.3)	31 (18.9)	<.001
Bothered by thinking or dreaming of terrible things, No. (%)	45 (6.7)	23 (14.0)	0.003
Brief symptom inventory, 6-item, mean (SD)	0.37 (1.48)	1.08 (2.91)	0.002
Previous diagnosis of major depression, No. (%)	15 (2.2)	22 (13.4)	<.001
Previous diagnosis of anxiety disorders, No. (%)	5 (0.7)	2 (1.2)	0.90
Ever had a problem with depression, No. (%)	58 (8.6)	55 (33.6)	<.001
Ever had a problem with anxiety, No. (%)	18 (2.7)	11 (6.7)	0.02
Talking to someone about your depression, No. (%)	15 (2.2)	25 (15.2)	<.001
Number of stressors, mean (SD)	1.85 (1.96)	3.03 (2.41)	<.001
Diabetes emotional burden, mean (SD)	2.32 (1.79)	3.66 (2.06)	<.001
Diabetes regimen distress, mean (SD)	2.22 (1.77)	3.46 (2.08)	<.001
Unemployed, No. (%)	416 (61.6)	114 (69.5)	0.07
Doing work for extra income, No. (%)	608 (90.1)	152 (92.7)	0.38
No health insurance, No. (%)	53 (7.9)	12 (7.3)	0.95

Feeling that my financial situation is getting worse, No. (%)	187 (27.7)	76 (46.3)	<.001
Having difficulty in paying bills, No. (%)	423 (62.7)	133 (81.1)	<.001
Having money left over at the end of the month, No. (%)	607 (89.9)	158 (96.3)	0.01
Financial worry score, mean (SD)	3.56 (2.08)	4.31 (2.02)	<.001
Sheehan disability scale, mean (SD)	0.90 (1.82)	2.55 (2.69)	<.001
Clinical variables			
Hemoglobin A1c, mean (SD), %	9.24 (2.14)	8.99 (2.03)	0.17
Body mass index, mean (SD), kg/m^2	32.53 (7.03)	33.90 (8.19)	0.05
Number of diabetes complications, mean (SD)	1.02 (1.06)	1.49 (1.06)	<.001
Taking insulin, No. (%)	348 (51.6)	94 (57.3)	0.22
On diabetes treatment – oral medication, No. (%)	581 (86.1)	148 (90.2)	0.20
On diabetes treatment – nutritionist observation, No. (%)	21 (3.1)	8 (4.9)	0.38
Had microalbumin test done in past 6 months, No. (%)	499 (73.9)	125 (76.2)	0.61
Taking pain medication, No. (%)	79 (11.7)	44 (26.8)	<.001
Taking antidepressant, No. (%)	18 (2.7)	30 (18.3)	<.001
Taking anxiety medication, No. (%)	3 (0.4)	3 (1.8)	0.17
Number of ICD-9 diagnoses in past 6 months, mean (SD)	6.75 (4.18)	8.12 (4.74)	<.001
Hospitalized overnight in past 6 months, No. (%)	84 (12.4)	28 (17.1)	0.15
ICU admitted in past 6 months, No. (%)	12 (1.8)	5 (3.0)	0.47
ER use in past 6 months, No. (%)	151 (22.4)	55 (33.5)	0.004
Number of primary care visits in past 6 months, mean (SD)	8.97 (5.94)	9.84 (5.97)	0.10
Had missed medical appointment in past 6 months, No. (%)	61 (9.0)	23 (14.0)	0.08
Future health care cost, mean (SD)	6354.50 (3524.89)	7418.23 (3725.23)	0.001
Enrolled into disease management program, No. (%)	468 (69.3)	112 (68.3)	0.87
Receiving automatic telephone screening and monitoring, No. (%)	237 (35.1)	46 (28.0)	0.10

[a]Patients who were later depressed were defined by DCAT study samples who had a baseline PHQ-2 score less than 3 and at least one PHQ-9 score greater than or equal to 10 at either 6, 12, or 18 months
